# Clinical Characteristics, Microbiological Spectrum, Biomarkers, and Imaging Insights in Acute Pyelonephritis and Its Complicated Forms—A Systematic Review

**DOI:** 10.3390/medicina62010222

**Published:** 2026-01-21

**Authors:** Marius-Costin Chițu, Teodor Salmen, Paula-Roxana Răducanu, Carmen-Marina Pălimariu, Bianca-Margareta Salmen, Anca Pantea Stoian, Viorel Jinga, Dan Liviu Dorel Mischianu

**Affiliations:** 1 Doctoral School, “Carol Davila” University of Medicine and Pharmacy, 020021 Bucharest, Romania; marius-costin.chitu@drd.umfcd.ro (M.-C.C.); bianca-margareta.mihai@drd.umfcd.ro (B.-M.S.); 2 Infectious Disease Department, Central Military Emergency University Hospital “Dr. Carol Davila”, 010825 Bucharest, Romania; carmen.palimariu@rez.umfcd.ro; 3 Cantacuzino National Military Medical Institute for Research and Development, 050096 Bucharest, Romania; 4 Pitesti County Emergency Hospital, 110084 Arges, Romania; 5 “Prof. Dr. Agrippa Ionescu” Emergency Clinical Hospital, 011356 Bucharest, Romania; paula0107@yahoo.com; 6 Department of Diabetes, Nutrition and Metabolic Diseases, Carol Davila University of Medicine and Pharmacy, 050474 Bucharest, Romania; anca.stoian@umfcd.ro; 7 Department of Urology, “Prof. Dr. Theodor Burghele” Clinical Hospital, 061344 Bucharest, Romania; viorel.jinga@umfcd.ro; 8 Department of Urology, University of Medicine and Pharmacy “Carol Davila” Bucharest, 020021 Bucharest, Romania; dan.mischianu@umfcd.ro; 9 Academy of Romanian Scientists, 050045 Bucharest, Romania

**Keywords:** acute pyelonephritis, obstructive pyelonephritis, biomarkers, procalcitonin, presepsin, computed tomography, antimicrobial resistance, urosepsis, systematic review, clinical, biological, radiological and/or microbiological

## Abstract

*Background and Objectives:* Acute and obstructive pyelonephritis (AOP) management, despite advancements in diagnostic imaging and antimicrobial therapy, is characterized by delayed recognition and increasing antimicrobial resistance. This review aimed to summarize current evidence regarding the clinical characteristics, microbiological spectrum, biomarkers, and imaging findings associated with AOP. *Materials and Methods:* A systematic review was conducted according to PRISMA guidelines and registered in PROSPERO (CRD420251162736). Literature searches were performed across the PubMed, Scopus, and Web of Science databases for articles published between January 2014 and 31 March 2025 using the term “acute obstructive pyelonephritis”. Inclusion criteria comprised original full-text English-language studies, published in the last 10 years and conducted in adults, reporting clinical, laboratory, microbiological, and imaging characteristics. Exclusion criteria are letters to the editor, expert opinions, case reports, conference or meeting abstracts, reviews, and redundant publications; having unclear or incomplete data; and being performed on cell cultures or on mammals. The quality of included studies was assessed using the Newcastle–Ottawa Scale. *Results:* Twenty-three studies met the inclusion criteria. AOP predominantly affected elderly patients with comorbidities, especially diabetes mellitus and urinary tract obstruction. Predictors of septic shock included thrombocytopenia, hypoalbuminemia, elevated procalcitonin (>1.12 µg/L), presepsin, and a neutrophil-to-lymphocyte ratio ≥ 8.7. *Escherichia coli* remained the leading pathogen (60–95%) with extended-spectrum β-lactamase (ESBL) rates between 20 and 70%, followed by *Klebsiella pneumoniae*. CT demonstrated 71–100% sensitivity for detecting obstructive complications, confirming its superiority over ultrasound, while MRI provided comparable diagnostic accuracy in selected cases. Source control through double-J stenting or percutaneous drainage significantly improved survival. *Conclusions:* AOP requires prompt recognition and early decompression to prevent sepsis-related mortality. Biomarkers such as procalcitonin, presepsin, and neutrophil to lymphocyte ratio enhance risk stratification, while CT remains the gold-standard imaging modality. The increasing prevalence of ESBL-producing pathogens underscores the need for antimicrobial stewardship and individualized therapeutic strategies guided by local resistance data.

## 1. Introduction

Urinary tract infections (UTIs) represent one of the most prevalent bacterial infections. They are encountered both in community and healthcare settings. Their management involves complex multidisciplinary medical teams [1,2,3].

Acute uncomplicated UTIs encompass both acute cystitis and acute pyelonephritis, clinical syndromes that occur in the absence of underlying renal or urological comorbidities, as stated by the definition proposed in 1992. If, in an acute UTI, signs that the infection extends beyond the bladder are present, we use the term complicated UTI to emphasize the association of any of the following symptoms: fever (>37.7 °C), chills, marked physical asthenia, inappetence, flank pain, costovertebral angle tenderness, and, for males, pelvic or perineal pain, suggestive of concomitant prostatitis [4]. In cases of pre-existing urological conditions (such as nephrolithiasis, urethral strictures, in-dwelling stents, or urinary diversions), associated with immunosuppressive states (including neutropenia or advanced HIV infection), or with poorly controlled diabetes mellitus (DM), they not necessarily classify as complicated UTIs, because it does not increase the risk for treatment failure, but for a slower response to treatment, depending on each patients reactivity [5,6,7,8,9]. Since 2025, the European Association of Urology Guidelines recommends abandoning the older terms of complicated and uncomplicated, and adopting UTIs’ division into systemic and localized, while maintaining the defining elements of the old classifications, because they consider the latter to be clearer and more useful for clinical and therapeutic decisions [10].

The incidence of UTIs increases progressively by age with no regard to gender, with the highest risk among sexually active premenopausal females [11,12]. Additional risk factors for uncomplicated UTIs include the use of specific contraceptive methods (notably spermicides), the presence of vaginal infections, DM, and obesity [13,14,15].

Due to the heterogeneous data available about UTIs, this systematic review aims to consolidate and present the existing knowledge, respectively, to evaluate clinical presentation, biological, radiological, and/or microbiological diagnostic characteristics along with monitoring and therapeutic tools, in order to improve UTI management in acute pyelonephritis (APN).

## 2. Materials and Methods

A systematic review was performed according to the guidelines and recommendations from the Preferred Reporting Items for Systematic Reviews and PRISMA 2020 Checklist (Table S1). The protocol for this review has been registered with the identifier CRD420251162736.

### 2.1. Research Question and Search Strategy

An electronic search for relevant publications was performed using the PubMed, Scopus, and Web of Science library databases from 1 January 2014 to 31 March 2025. The following search strategy was used: “acute obstructive pyelonephritis” for each of the three databases. In this search, 1518 articles were found (656 from PubMed, 525 from Scopus, and 337 from Web of Science). We eliminated 98 duplicates and remained with 1420 studies. After applying filters in each of the three databases’ filters for language (English), publication type (original articles), and date range (2014 to the date of the search), 269 articles remained. From the remaining 269 articles, case reports and studies on infant or pediatric populations were eliminated, resulting in 29 articles that underwent initial title screening, followed by abstract review by two independent reviewers. After excluding the articles that did not meet the inclusion criteria, 20 articles remained for full assessment. We included 3 more articles identified when we searched the references of the relevant studies, leading to a total of 23 included articles.

The research question was framed using the Population, Intervention, Comparison, and Outcome (PICO) method. The population was represented by patients with complicated UTIs treated incompletely or incorrectly with antibiotics, which have progressed to acute obstructive pyelonephritis. The outcome was defined by clinical, biological, radiological, and/or microbiological characteristics of acute and obstructive pyelonephritis (AOP), with effect measured by percentage, confidence interval (CIs), odds, or relative risks and correlation levels.

### 2.2. Inclusion Criteria

To be included in this systematic review, articles had to meet the following publication criteria: (1) articles published in English; (2) original full-text articles with cohort and cross-sectional studies; (3) conducted on adult human populations; (4) published in the last ten years; (5) articles with patients with complicated UTIs treated incompletely or incorrectly with antibiotics, have microbiological data recorded and that may develop AOP; (6) articles with sufficient data provided, such as appropriate 95% CIs, *p*-values, or correlations with various risk factors and markers of disease severity.

### 2.3. Exclusion Criteria

Studies were excluded from the analysis if they were (1) letters to the editor, expert opinions, case reports, conference or meeting abstracts, or reviews; (2) redundant publications; (3) presenting unclear or incomplete data; (4) performed on cell cultures or on mammals; (5) not identifying a characteristic of interest—clinical, biological, radiological and/or microbiological; or (6) providing imprecise or incomplete data.

### 2.4. Data Extraction

Two authors used a self-made data extraction table to individually evaluate and extract the following data for each included literature reference: the reported characteristic—clinical, biological, radiological, and/or microbiological—with their outcomes as 95% CIs or mean values. Any differences of opinion were settled through discussion or consultation with a third author.

### 2.5. Risk of Bias Assessment

Two reviewers independently assessed the quality of the studies using the Newcastle–Ottawa Scale (NOS), a star rating system that evaluates articles on selection, comparability, and outcome criteria [16]. Research papers rated with at least six stars, as evaluated on the NOS, are considered of good quality and included in the Results Section. This selection process is summarized in Table 1 for cohort studies and in Table 2 for cross-sectional studies.

### 2.6. Strategy for Data Synthesis

A narrative synthesis of the findings in the studies is centered around the clinical, biological, radiological, and/or microbiological characteristics of acute obstructive pyelonephritis. The studies are heterogeneous (study design, study quality, screening methods described, interventions, and outcomes). Therefore, a narrative synthesis was performed using text and tables to provide a descriptive summary and explanation of study characteristics and findings.

## 3. Results

**Figure 1:**
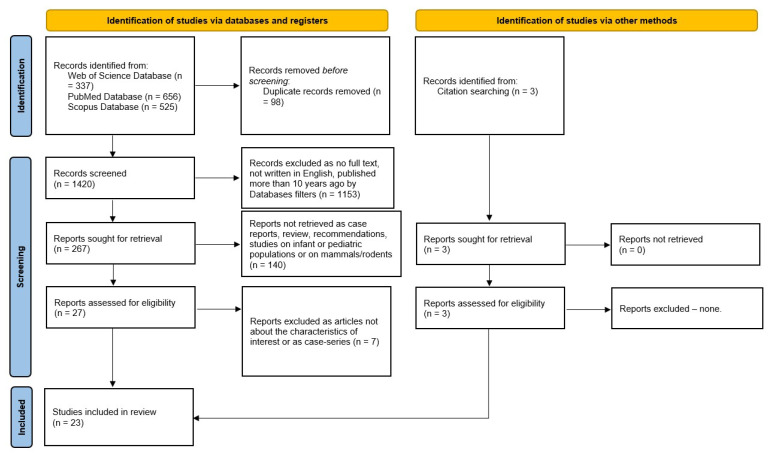
Flowchart of the study selection process.

This systematic review incorporates twenty-three studies published between 2014 and 2025, as shown in Figure 1.

The clinical characteristics and risk factors in APN/AOP from the included studies are summarized in Table 3 and Table 4.

The clinical characteristics of APN are fever, flank pain, and laboratory-confirmed pyuria and bacteriuria at threshold-dependent colony counts, with blood cultures remaining essential in complicated or obstructive cases. Risk factors are more pronounced among elderly or patients with comorbidities, in whom atypical presentations, higher bacteremia, extended-spectrum β-lactamase (ESBL) rates, and predictors such as DM or thrombocytopenia necessitate early recognition and timely decompression to reduce sepsis-related morbidity and mortality.

Laboratory parameters are summarized in Table 5.

Relevant laboratory biomarker levels in AOP are, particularly, elevated procalcitonin (PCT), presepsin (PSEP), and Neutrophil-To-Lymphocyte Ratio (NLR), alongside thrombocytopenia and hypoalbuminemia. These serve as key independent predictors of severity, sepsis, and septic shock in APN and AOP, with combined PCT–PSEP–NLR assessment outperforming conventional parameters such as C-reactive protein (CRP) or leukocytosis and enabling more accurate early risk stratification, especially in elderly or patients with comorbidities and even in resource-limited clinical settings.

The main causative agents of UTIs and their clinical implications regarding recurrent infections, antibiotic use, and healthcare institutions are summarized in Table 6.

The main microbiological spectrum is characterized by *Escherichia coli* (*E. coli*), which remains the dominant uropathogen in APN and AOP but with increasingly high ESBL and multi-drug-resistant (MDR) rates—manifested by extensive cross-resistance to fluoroquinolones and cephalosporins and a broader, more heterogeneous microbial spectrum in complicated infections—thereby underscoring the necessity for individualized empirical therapy guided by local resistance profiles, preserved susceptibility to agents such as piperacillin–tazobactam and aminoglycosides, and heightened vigilance in elderly or septic patients who disproportionately harbor MDR organisms.

The most used imaging instruments and their specificity and prognostic significance in UTI are summarized in Table 7.

The studies demonstrate that while US provides accessible first-line screening with moderate sensitivity for detecting APN/AOP, contrast-enhanced CT remains the diagnostic gold standard and most accurate modality for identifying parenchymal and perirenal complications—including stones, abscesses, gas, and emphysematous changes—whereas MRI offers CT-comparable performance and superior lesion characterization in patients who cannot receive iodinated contrast, collectively supporting a tiered imaging strategy tailored to disease severity and clinical context.

The main therapeutic interventions and their outcome in the management of APN/AOP are summarized in Table 8.

## 4. Discussion

Our study analyzed the available data in the specialized literature regarding the clinical, biological, radiological, and/or microbiological characteristics of acute complicated and uncomplicated UTIs, in order to improve case management in medical practice.

### 4.1. Clinical Parameters

The diagnostic criteria for APN have evolved, yet they generally rely on a combination of clinical and laboratory parameters. APN is typically defined by the presence of fever ≥38 °C, flank pain or costovertebral angle tenderness, and laboratory evidence of pyuria and bacteriuria. Pyuria is commonly defined as ≥5–10 leukocytes per high-power field in urine sediment, while significant bacteriuria is considered at thresholds of ≥10^4^–10^5^ Colony-forming Unit (CFU)/mL, depending on gender, method of urine collection, and the presence of complicating factors. In males, ≥10^4^ CFU/mL is generally considered significant, whereas in females, thresholds vary from ≥10^3^ CFU/mL in acute uncomplicated cystitis to ≥10^5^ CFU/mL in complicated UTIs [5,6,7].

The European Association of Urology Guidelines further refine these criteria, recommending colony counts of ≥10^3^ CFU/mL in symptomatic females with acute uncomplicated cystitis, ≥10^4^ CFU/mL in pyelonephritis, and ≥10^5^ CFU/mL in complicated UTIs. In catheterized samples, even 10^2^ CFU/mL may represent true infection. Even though, in some complicated pyelonephritis, a germ cannot be identified in the urine, the management of uncomplicated UTIs does not require blood culture to guide empirical antibiotic therapy, but in pyelonephritis complicated by/with bacteremia/sepsis, blood cultures, even less sensitive ones, remain the gold standard for detecting bacteremia and guiding antimicrobial therapy [8,9,10].

AOP is diagnosed when APN coexists with urinary tract obstruction, most often due to calculi. Clinical manifestations may overlap with uncomplicated APN, but obstruction significantly increases the risk of sepsis and mortality.

The present study identified that APN predominantly affects young, sexually active females, while AOP is more prevalent among elderly individuals and those with comorbidities such as DM or urinary obstruction. These findings align closely with Chang et al. [35], who reported that older females with community-acquired APN exhibited higher rates of bacteremia, elevated CRP, and ESBL infections compared with younger cohorts. Similarly, Lee et al. [28] demonstrated that atypical presentations such as confusion, oliguria, or general malaise were predominant among elderly patients with obstructive urolithiasis, contributing to diagnostic delays and higher sepsis rates. The current data also confirm that DM and thrombocytopenia are strong predictors of septic shock, in agreement with Yamamichi et al. [33] and Kakinoki et al. [40], who emphasized the need for early decompression in obstructed cases. Collectively, these parallels underscore the consistency of the present results with international trends, supporting early recognition and timely intervention as crucial determinants of survival [13,20,21,22,23,25].

### 4.2. Biological and Biomarker Parameters

The studies highlight the prognostic importance of proinflammatory biomarkers in assessing the severity of AOP and APN, including PCT > 1.12 µg/L, PSEP, NLR ≥ 8.7, thrombocytopenia, and hypoalbuminemia. Similar data were reported by Baboudjian et al. [25], who demonstrated that PCT is an early predictor of septic complications, with superior performance to CRP and leukocytosis. In a complementary manner, Tambo et al. [27] confirmed the independent role of PSEP and PCT in predicting sepsis and septic shock, emphasizing the faster dynamics of PSEP, but also the limitations of its interpretation in patients with renal dysfunction.

The role of NLR as an accessible and cost-effective marker has been validated by several studies [17,18], which associated it with an increased risk of septic shock and prolonged hospitalization duration. Converging literature suggests that the combined use of PCT, PSEP, and NLR improves the accuracy of risk stratification, especially in settings with limited laboratory resources. Overall, PCT and PSEP are distinguished by superior diagnostic and prognostic performance, while CRP remains a nonspecific marker [7,9,12,23,27,33].

Several independent risk factors for progression to sepsis and septic shock have been consistently identified in APN and AOP. DM, through mechanisms such as glycosuria, impaired immunity, and urinary stasis, increases the likelihood of developing septic shock by up to fivefold [6]. Thrombocytopenia (<150 × 10^9^/L) and hypoalbuminemia (<3.0 g/dL) have been shown to be independent predictors of severity, reflecting both immune dysfunction and increased vascular permeability [7,11]. NLR ≥ 8.7 has a high sensitivity for identifying high-risk patients [7,11,41], and PCT and PSEP clearly outperform CRP and WBC in estimating the probability of sepsis, septic shock, need for intensive care admission, and mortality, with PSEP having an earlier onset of increase, but with limitations in patients with advanced chronic kidney disease [11,12,41]. Other parameters, such as leukocytosis, CRP, or creatinine, although frequently altered, do not demonstrate independent predictive value after multivariate adjustment.

In the context of risk stratification, biomarkers have become essential tools in the management of APN and AOP. CRP, although widely used, has low specificity in predicting sepsis [7,13,14,15]. PCT is distinguished by its superior accuracy—correlating with disease severity, length of hospitalization duration, and postoperative complications [11,12,14]—and PSEP, detectable early and correlated with the degree of acute kidney injury, is emerging as a promising biomarker, despite the influence of renal clearance on its values [12,26]. NLR remains an additional inflammatory marker, especially useful in elderly patients and in situations where the availability of advanced tests is limited [7,11]. The combination of PCT–PSEP–NLR provides the best performance in identifying high-risk patients who require rapid intervention and intensive monitoring.

### 4.3. Microbiological Spectrum and Antimicrobial Resistance

The current findings reveal *E. coli* as the predominant pathogen (60–95%) in both APN and AOP, with high rates of ESBL production (20–70%) and cross-resistance to fluoroquinolones and third-generation cephalosporins. These results correspond to those of Jang et al. [23], who observed higher resistance rates among isolates from obstructive pyelonephritis compared to uncomplicated cases, and to Hyun et al. [29], who reported that empirical adequacy was significantly higher for *Klebsiella pneumoniae* (96%) than for *E. coli* (70.6%), reflecting greater resistance heterogeneity. The increasing prevalence of MDR organisms was also noted by Cornejo-Dávila et al. [36] and Yamamichi et al. [33], underscoring the global shift toward carbapenem or β-lactam/β-lactamase inhibitor combinations as first-line empirical regimens in severe infections. The present study’s observation of persistently low resistance to piperacillin–tazobactam and aminoglycosides remains consistent with contemporary stewardship recommendations (Tamma et al., [41]). These comparisons collectively highlight the urgent need for individualized antimicrobial strategies guided by local resistance profiles [9,19,21,29,30,40,41].

*E. coli* remains the predominant uropathogen in both APN and AOP, responsible for 60–95% of community-acquired infections. Non-*E. coli Enterobacteriaceae*, such as *Klebsiella pneumoniae*, *Proteus mirabilis*, *Enterobacter cloacae*, and *Citrobacter* spp., account for a smaller proportion but are clinically significant due to their frequent antimicrobial resistance. Other pathogens include *Enterococcus faecalis*, *Staphylococcus aureus*, *Staphylococcus saprophyticus*, and *Pseudomonas aeruginosa*, with fungi such as *Candida albicans* occasionally implicated in catheterized patients or those exposed to broad-spectrum antibiotics [6,8,10,11,12].

The microbial spectrum of complicated UTIs, including AOP, is broader and more heterogeneous compared to uncomplicated infections. European multicenter studies confirm *E. coli* as the leading pathogen across urologic departments, but with a growing proportion of non-*E. coli* isolates and MDR organisms, particularly in hospitalized patients. In intensive care settings, the distribution of pathogens is more diverse, with *E. coli*, *Candida* spp., *Enterococcus* spp., *P. aeruginosa*, and *Klebsiella* spp. frequently encountered [5,8,13].

### 4.4. Antimicrobial Resistance Patterns

The rise of antimicrobial resistance, especially ESBL-producing *Enterobacteriaceae*, significantly impacts clinical management. Studies reveal ESBL-producing *E. coli* rates ranging from 20% to over 70% in certain cohorts, with alarming cross-resistance to fluoroquinolones (nearly universal in some series), cephalosporins, and trimethoprim–sulfamethoxazole. Resistance to aminoglycosides and nitrofurantoin remains lower, though still clinically relevant. Carbapenem resistance, while less prevalent, has been reported and represents a critical therapeutic concern [6,12,13,14].

The elderly population appears disproportionately affected by ESBL infections, correlating with prior antibiotic use, recurrent UTIs, and comorbidities. Moreover, septic patients are more likely to harbor MDR pathogens compared to non-septic individuals [6,13,30,35].

Large-scale epidemiological data from over 205 million emergency department visits demonstrated that cystitis accounted for 4.3% of cases (n = 8,768,481) and pyelonephritis for 0.5% (n = 1,044,742). Admission rates were 23.9% for cystitis and 33.4% for pyelonephritis, with a rising trend over time. Antibiotic prescribing patterns showed increased reliance on third- and fourth-generation cephalosporins for admitted patients, while fluoroquinolone use declined [28]. In contrast, discharged patients were more frequently prescribed first-generation cephalosporins, nitrofurantoin, or trimethoprim–sulfamethoxazole, though use of these agents has also declined in recent years [9,14,42].

### 4.5. Imaging Findings

Imaging findings in the present study showed that ultrasonographic abnormalities were identified in 57.9% of patients, with the prominent renal pyramid sign appearing in approximately one-quarter of cases. The higher detection rates of CT, exceeding 70%, align with previous literature identifying CT as the reference standard for assessing parenchymal and perirenal complications [33,39]. MRI findings demonstrated diagnostic performance comparable to CT, particularly in differentiating purulent from cystic lesions when contrast administration is contraindicated. Imaging features such as stones > 5 mm and the presence of gas strongly correlated with septic progression, reinforcing their prognostic significance [33,43]. Overall, these data support a tiered imaging strategy: ultrasound (US) as an initial screening tool, CT for definitive assessment, and MRI as an adjunct in selected scenarios [15,21,28,31,33,34,35,38,44].

*US* remains the first-line modality in suspected APN due to its accessibility, bedside applicability, and absence of ionizing radiation. On B-mode, structural abnormalities are observed in more than half of patients, with the prominent renal pyramid sign indicating medullary edema, while parenchymal thickening and reduced renal mobility further reflect active inflammation. However, a substantial proportion of patients may exhibit normal US findings in early disease, underscoring the limited sensitivity of the technique as a standalone diagnostic tool [7,14].

Color Doppler enhances diagnostic accuracy by assessing renal perfusion. Diffuse hyperemia—described as the “flaring kidney sign”—suggests acute inflammatory activity, whereas focal avascular regions raise suspicion for necrosis or abscess formation and assist in directing clinical management [15,34].

Complicated forms, including renal abscesses and carbuncles, appear as well-defined hypoechoic lesions with absent Doppler flow, consistent with suppurative destruction [44]. In obstructive pyelonephritis, US readily identifies pelvicalyceal dilation and features of pyonephrosis, such as echogenic debris or reverberation artifacts from gas. Nevertheless, a normal US cannot reliably exclude obstruction, particularly in early stages or cases of functional obstruction [45].

Although US is valuable for rapid initial evaluation, especially in pregnant or hemodynamically unstable patients, its operator dependence and limited ability to detect subtle parenchymal changes restrict its role in comprehensive assessment [35].

*Contrast-enhanced CT* is the most accurate imaging modality for diagnosing APN and AOP, providing high-resolution visualization of the renal parenchyma, collecting system, and perirenal tissues, and enabling reliable detection of complications.

Typical APN findings include renal enlargement, parenchymal thickening, and reduced corticomedullary differentiation. More specific features consist of hypodense wedge-shaped or triangular areas representing edema, microvascular spasm, and tubular obstruction [39].

In complicated disease, CT accurately identifies renal abscesses, carbuncles, and intrarenal or collecting-system gas, the hallmark of emphysematous pyelonephritis, a life-threatening emergency. Perinephric fat stranding, commonly observed, correlates with extensive inflammation and severe clinical presentation [43,44].

In obstructive pyelonephritis, CT detects ureteral or renal calculi with up to 97% sensitivity and differentiates infected from noninfected obstruction. Pelvicalyceal wall thickening and gas within the collecting system in the absence of instrumentation strongly indicate infected fluid. The striated nephrogram pattern—alternating bands of cortical enhancement—is seen in both APN and pyonephrosis and is typically more pronounced in the latter [34].

The sensitivity of CT for detecting APN, abscesses, and emphysematous changes ranges between 71.7% and 100%. Early inflammatory lesions often resolve completely following antibiotic therapy, without residual scarring [11,12,14,35,37].

*MRI*, although less frequently used, provides superior soft-tissue contrast and avoids ionizing radiation, making it suitable when CT is contraindicated, such as during pregnancy, iodinated contrast allergy, or advanced renal insufficiency.

MRI features of APN include renal enlargement, parenchymal thickening, and reduced corticomedullary differentiation. On T2-weighted images, lesions appear as poorly demarcated hyperintense areas, while T1-weighted sequences typically show hypointense regions, reflecting edema and inflammatory infiltration [39,43].

MRI reliably differentiates renal abscesses from infected cysts by characterizing intracystic fluid composition—a distinction essential for guiding therapeutic decisions. Comparative studies demonstrate diagnostic performance equivalent to CT in detecting infiltrative and purulent lesions, positioning MRI as a valuable alternative when CT cannot be performed [31].

### 4.6. Radiological Predictors of Obstructive Pyelonephritis

Specific radiological features have been associated with obstructive pyelonephritis and poor outcomes:

*Stone diameter:* Stones > 5 mm, especially in the ureters, strongly correlate with obstructive pyelonephritis, regardless of total stone burden. Previous studies reported mean stone diameters around 10 ± 8 mm [35].

*Hydronephrosis:* Universally present in AOP, reflecting obstruction of the collecting system.

*Perirenal fat stranding*: Associated with higher CRP levels, fever, and pyuria, and should be considered an early radiological indicator of significant parenchymal inflammation.

*Gas formation:* The presence of intrarenal or perirenal gas in the absence of recent instrumentation is highly specific for emphysematous pyelonephritis [15].

### 4.7. Comparative Overview Table

Table 9 summarizes a comparative overview of the results.

### 4.8. Complicated APN, Including AOP

Management of complicated APN, particularly in cases associated with obstruction, abscess formation, or emphysematous changes, frequently requires urological intervention in addition to antimicrobial therapy. AOP shares similar infectious mechanisms and microbiological profiles, leading to increased intrarenal pressure, altered bacterial clearance, and reduced antibiotic penetration, with the main differences summarized in Table 10.

So, AOP is a urological emergency, with a higher risk of sepsis and renal damage, requiring urgent relief of obstruction prior to antimicrobial treatment. Bilateral double-J (DJ) stenting was the most commonly performed procedure, applied in 85 patients (76.6%) with APN and in 62 patients (32.6%) with upper urinary tract calculi. Percutaneous nephrostomy was performed in selected cases of emphysematous pyelonephritis (n = 3) and pyonephrosis (n = 4). For perinephric abscesses, percutaneous drainage was preferred (77.8%), whereas open drainage was used in 22.2% of cases. Renal abscesses were predominantly managed with percutaneous drainage (75%), while open drainage combined with DJ stenting was reserved for refractory cases.

Lower urinary tract complications were addressed through suprapubic catheterization, particularly in patients with urethral strictures (n = 49) or prostatic abscesses requiring deroofing (n = 33). Orchiectomy was indicated in 14 patients due to testicular abscesses (n = 8) or malignancy (n = 6). Additional procedures included cystolitholapaxy in 11 patients with bladder calculi [8,11].

### 4.9. Drainage Methods

AOP represents one of the most critical urological emergencies, requiring prompt recognition and intervention due to its rapid progression toward sepsis and multi-organ dysfunction. The combination of UTI and mechanical obstruction creates a high-pressure infected system that necessitates immediate decompression to prevent systemic deterioration. Recent comparative research continues to refine our understanding of optimal management pathways, including the choice of drainage technique and the clinical impact of intervention timing. The expanding body of evidence highlights the importance of individualized approaches based on hemodynamic stability, anatomic factors, and resource availability [46,47,48,49].

Percutaneous nephrostomy (PCN) and DJ retrograde ureteral stenting remain the principal modalities for urgent decompression in AOP, each offering distinct clinical advantages depending on the presentation. PCN provides direct access to the renal collecting system and is often preferred when retrograde access is technically challenging, such as in severe edema, anatomical abnormalities, or hemodynamic instability. Conversely, DJ stenting is advantageous in situations where endoscopic access is feasible and immediate operating room availability permits rapid intervention. Additional emerging techniques, including US bedside PCN in the emergency setting, have expanded access to rapid decompression for unstable patients [46,47,48,49,50].

Large-scale analyses and meta-analyses consistently show that PCN and DJ stenting achieve comparable efficacy in controlling infection and restoring urinary flow. While immediate clinical outcomes—including fever resolution, leukocyte normalization, and overall rates of septic improvement—appear equivalent between techniques, nuanced differences still exist. For example, some studies report a slightly faster improvement in renal function following PCN, whereas others indicate greater patient comfort with PCN due to the absence of stent-related lower urinary tract symptoms. Furthermore, although complication rates are similar, PCN may result in minor tube-related issues, and DJ stents may require subsequent procedures if obstruction recurs or stent migration occurs [47,49].

The timing of intervention remains a decisive factor in patient prognosis, particularly in cases complicated by systemic inflammatory response or septic shock. Evidence suggests that delays in decompression—typically beyond the first several hours—are associated with prolonged hospitalization, higher rates of intensive-care units’ admission, and increased risk of organ dysfunction. Early drainage, regardless of method, appears consistently beneficial in halting septic progression and improving hemodynamic stabilization. Some observational studies also suggest that delayed decompression may adversely affect long-term renal function, further underscoring the urgency of early intervention in AOP [47,48].

Clinical decision-making in AOP should integrate both patient-specific and system-level factors to ensure optimal outcomes. In unstable or severely septic individuals, PCN may be the preferred choice due to its feasibility at the bedside and minimal need for anesthesia. Conversely, DJ stenting is a strong option for stable patients when retrograde access is anticipated to be straightforward and when immediate endoscopic resources are available. Ultimately, the consistent message across current literature is the primacy of rapid decompression; once drainage is secured, antimicrobial therapy and supportive measures form the foundation of recovery. Increasingly, multidisciplinary coordination between urology, emergency medicine, and critical care is recognized as essential in optimizing management pathways [46,47,48].

Despite significant advancements in understanding AOP management, current evidence is limited by the predominance of retrospective and observational designs. Variability in patient selection, definitions of severe infection, timing thresholds, and outcome measures complicate direct comparisons between studies. Moreover, inherent procedural selection bias—where sicker patients may preferentially receive one technique over another—limits the strength of causal inferences. Future research would benefit from well-designed randomized controlled trials, standardized outcome definitions, and multicenter collaboration to clarify optimal intervention timing and the nuanced advantages of each drainage modality [46,47,48,49,50].

### 4.10. Supportive Management in Urosepsis

In patients with urosepsis, timely supportive measures are as critical as antimicrobial therapy. Early fluid resuscitation with crystalloids at 30 mL/kg is recommended, followed by prompt initiation of vasopressor support to maintain a mean arterial pressure > 65 mmHg, with norepinephrine as the first-line agent. Glycemic control with insulin therapy, corticosteroid administration in refractory shock, and transfusion of blood products were indicated as essential adjuncts of care [13].

### 4.11. Duration of Antimicrobial Therapy

Evidence supports the safety and efficacy of shorter antibiotic courses in APN. A randomized trial demonstrated that discontinuation of non-fluoroquinolone regimens at Day 7, once clinical improvement was sustained, did not increase relapse rates compared with 14-day therapy. This aligns with meta-analyses showing equivalence between ≤7 days and longer regimens in terms of clinical and microbiological failure, even in patients with bacteremia. Historical data on aminoglycosides suggest bacteriological cure rates exceeding 90% with 5-day regimens, though subsequent reviews indicated inferiority compared with β-lactams or fluoroquinolones. Nevertheless, aminoglycosides remain viable in specific settings, particularly when combined with β-lactams [8,11].

### 4.12. Limitations

This review is limited by the heterogeneity of included studies, variations in study design, and the lack of quantitative meta-analysis.

Even if the main body, more than 86.5%, of medical literature is published in English—the lingua franca of scientific communications—including only studies published in English could be a potential limitation [51].

Some data were derived from retrospective cohorts with incomplete microbiological or biomarker reporting.

The predominance of single-center studies and geographic variability in antimicrobial resistance patterns may also restrict generalizability.

Future multicentric, prospective studies are required to validate biomarker thresholds and integrate imaging-based risk scoring systems.

Another important limitation that may be drawn is the aim to present mainly the medical aspects of AOP and not emphasize the surgical/urological aspects throughout.

## 5. Conclusions

AOP remains a medical and urological emergency requiring early recognition and multidisciplinary management.

Acute pyelonephritis (APN) can be defined as follows:

- Clinically—Characterized by fever ≥ 38 °C, flank pain or costovertebral angle tenderness, and a higher risk of severe or atypical presentations in elderly or patients with comorbidities (e.g., DM, thrombocytopenia), who are also more prone to septic progression and obstructive forms.
- Laboratory—Characterized by pyuria (≥5–10 WBC/HPF), significant bacteriuria at gender- and context-dependent thresholds (10^3^–10^5^ CFU/mL), and severity stratification supported by proinflammatory biomarkers such as elevated PCT, PSEP, NLR ≥ 8.7, thrombocytopenia, and hypoalbuminemia, with blood cultures essential in complicated, septic, or obstructive cases.
- Microbiologically—Characterized predominantly by *E. coli* (60–95%), frequently exhibiting ESBL production and MDR, with broader pathogen heterogeneity in complicated or obstructive disease (including *Klebsiella*, *Proteus*, *Enterobacter*, *Pseudomonas*, *Enterococcus*, *Candida*), necessitating individualized empirical therapy guided by local resistance patterns and preserved susceptibility to agents such as piperacillin–tazobactam and aminoglycosides.
- Imaging—Characterized by limited early sensitivity on US (though useful as first-line), definitive parenchymal and perirenal assessment on contrast-enhanced CT (detecting edema, hypoattenuating wedges, abscesses, gas, stones, and emphysematous changes), and MRI offering CT-comparable accuracy and superior soft-tissue characterization when contrast CT is contraindicated.

Further research should focus on developing predictive models that combine clinical, microbiological, and imaging data to optimize outcomes and limit antimicrobial overuse.

## Figures and Tables

**Table 1:** Newcastle–Ottawa Scale analysis of the cohort studies included [17,18,19,20,21,22,23,24,25,26,27,28,29,30,31,32,33,34,35,36,37].

Author (Reference)	Selection				Comparability	Outcome			Total Score	Quality
	Representativeness of the Exposed Cohort	Selection of the Non-Exposed Cohort	Ascertainment of Exposure	Demonstration That Outcome of Interest Was Not Present at Start of Study	Comparability of Cohorts Based on the Design or Analysis	Assessment of Outcome	Was Follow-Up Long Enough for Outcomes to Occur	Adequacy of Follow-Up of Cohorts		
Min et al. [17], 2024	+	-	+	+	+	+	+	+	7	Very good
Truong et al. [18], 2024	+	+	+	+	+	+	-	-	6	Good
Hayano et al. [19], 2024	+	+	+	+	+	+	-	-	6	Good
Yamamoto et al. [20], 2023	+	-	+	+	+	+	+	+	7	Very good
Murugan et al. [21], 2022	+	-	+	+	+	+	+	-	6	Good
Song et al. [22], 2022	+	+	+	+	-	+	+	-	6	Good
Jang et al. [23], 2022	+	-	+	+	+	+	+	-	6	Good
Ishikawa et al. [24], 2021	+	+	+	+	+	+	-	-	6	Good
Baboudjian et al. [25], 2021	+	-	+	+	+	+	+	-	6	Good
Stokes et al. [26], 2021	+	-	+	+	+	+	+	+	7	Very good
Tambo et al. [27], 2020	+	-	+	+	+	+	+	-	6	Good
Lee et al. [28], 2020	+	-	+	+	+	+	+	-	6	Good
Hyun et al. [29], 2019	+	+	+	+	-	+	+	-	6	Good
Pierce et al. [30], 2019	+	-	+	+	+	+	+	-	6	Good
Lee et al. [31], 2019	+	-	+	+	+	+	+	-	6	Good
Rudrabhatla et al. [32], 2018	+	-	+	+	+	+	+	+	7	Very good
Yamamichi et al. [33], 2018	+	-	+	+	+	+	+	-	6	Good
Enikeev et al. [34], 2017	+	+	-	+	+	+	+	-	6	Good
Chang et al. [35], 2015	+	+	+	-	+	+	+	-	6	Good
Cornejo-Dávila et al. [36], 2015	+	+	+	-	+	+	+	-	6	Good
Vahlensieck et al. [37], 2015	+	-	+	+	+	+	+	+	7	Very good

“+” indicates that the article meets the criteria mentioned above; “-” indicates that the article does not meet the abovementioned criteria.

**Table 2:** Newcastle–Ottawa Scale analysis of cross-sectional studies included [38,39].

Author (Reference)	Selection				Comparability	Outcome		Total Score	Quality
	Representativeness of the Cases	Sample Size	Non-Response Rate	Ascertainment of the Screening/Surveillance Tool	The Potential Confounders Were Investigated by Subgroup Analysis or Multivariable Analysis	Assessment of Outcome	Statistical Test		
Gottlieb et al. [38], 2025	+	+	+	+	+	+	-	6	Good
Abi Tayeh et al. [39], 2022	+	-	+	+	+	+	+	6	Good

“+” indicates that the article meets the criteria mentioned above; “-” indicates that the article does not meet the abovementioned criteria.

**Table 3:** Clinical characteristics and risk factors in APN/AOP.

Reference	Country	No. of Patients	Study Period	Clinical Parameter Evaluated	Results
Chang et al., 2015 [35]	South Korea	315	2015	Hospitalization days in community-onset APN	9 for elderly women versus 8 for non-elderly women
Murugan et al., 2022 [21]	India	82	2022	Clinical profile and outcome in urosepsis	12.5% mortality Comorbidities: diabetes mellitus, high blood pressure, chronic kidney disease.
Hyun et al., 2019 [29]	South Korea	229	2019	*K. pneumoniae* versus *E. coli* infections	More severe forms (66.7% vs. 70.4%) Delayed recovery (19 versus 14 days of hospital stay)
Cornejo-Dávila et al., 2015 [36]	Mexico	150	2015	Management of complicated UTIs in a tertiary center	53.2% of presentations as APN 71.4% ESBL—producing *E. coli*
Gottlieb et al., 2025 [38]	USA	NR	2016–2023	Epidemiology of UTIs in emergency departments	Steady rise in APN cases, with trend toward complicated forms

APN = acute pyelonephritis; AOP = acute and obstructive pyelonephritis; ESBL = extended-spectrum beta-lactamase; UTI = urinary tract infection; *E. coli* = *Escherichia coli*; *K. pneumoniae* = *Klebsiella pneumoniae*; NR—not reported.

**Table 4:** Clinical Characteristics and Risk Factors Levels in APN/AOP.

Parameter	Findings/Description	Comments/Clinical Significance
Predominant age groups [22,24,35]	Young sexually active women (uncomplicated APN) versus elderly population (AOP, higher severity)	Incidence and risk to develop bacteremia and severe outcomes increases with age
Typical symptoms (younger adults) [21,24,35]	Fever ≥ 38 °C, flank pain, costovertebral angle tenderness, dysuria, frequency	Classical triad in community-acquired APN
Atypical presentation (elderly) [21,22,24,35]	Confusion, oliguria, malaise, absence of flank pain	Leads to diagnostic delay and higher sepsis rates
Comorbidities associated with complications [21]	DM, urinary tract obstruction, immunosuppression, prior antibiotics	Independently associated with septic shock and mortality
Predictors of septic shock [24,25,26,27]	DM, thrombocytopenia, hypoalbuminemia, NLR ≥ 8.7, elevated PCT/PSEP	Independent prognostic markers
Clinical outcomes [17,18]	Higher bacteremia, ESBL infection, leukocytosis ≥ 15,000/mm^3^, CRP ≥15 mg/dL in elderly	Reflects systemic inflammation and advanced infection
Hospitalization and mortality [38]	Increased in AOP vs. APN; early obstruction relief crucial	Source control and rapid antimicrobial therapy improve outcomes

APN = acute pyelonephritis; AOP = acute and obstructive pyelonephritis; NLR = Neutrophil-to-Lymphocyte Ratio; PCT = procalcitonin; PSEP = presepsin; ESBL = extended-spectrum beta-lactamase; DM = Diabetes mellitus.

**Table 5:** Laboratory and biomarker Findings in APN/AOP.

Reference	Country	No. of Patients	Study Period	Biomarker/Score	Results
Baboudjian et al., 2021 [25]	France	126	2021	PCT > 2 ng/mL	Linked to septic shock and >10-day hospitalization
				PCT > 1.12 µg/L as prognostic value	Correlates with septic shock risk; Early rise
				PCT versus CRP	Sensitivity (95% versus 82%) Specificity (77% versus 66%) Superior in sepsis prediction
Tambo et al., 2020 [27]	Japan	104	2020	PSEP in sepsis prediction	88% sensitivity 78% specificity
				PSEP versus PCT	Rises faster in infection Corelates with AKI and sepsis Higher predictive for sepsis (*p* = 0.001 vs. *p* = 0.015)
Min et al., 2024 [17]	Turkey	47	2024	NLR, PLR	Cost-effective markers Elevated in severe sepsis NLR > 5.5 predictive of septic shock Independent predictors of severe sepsis in septic shock
				WBC ≥ 15,000/mm^3^	Frequent; nonspecific Not an independent predictor
Truong et al., 2024 [18]	Vietnam	68	2024	NLR, MLR, PLR	Significant increase in septic shock (12.5 times increase for NLR)
				NLR ≥ 8.7	Increase in septic shock Cost-effective marker Independent prognostic value
				Platelet count < 150 × 10^9^/L	Strongly associated with septic shock Independent predictor of poor outcome
				Serum albumin < 3.0 g/dL	Reflects inflammation and malnutrition Predicts septic shock and prolonged stay
Ishikawa et al., 2021 [24]	Japan	165	2021	qSOFA score ≥ 2	Predictive of mortality and multiorgan failure
					Increased mortality and prolonged hospitalization in elderly versus young patients with APN, but with lower sensitivity and specificity
Lee et al., 2020 [28]	South Korea	206	2020	SOFA score ≥ 5	Linked to higher mortality in obstructive cases
Murugan et al., 2022 [21]	India	82	2022	CRP, IL-6, leukocytosis	CRP > 100 mg/L associated with poor outcome
Yamamoto et al., 2023 [20]	Japan	63	2023	Urinary NGAL	Elevated in bacteremic APN; early marker of renal injury
				Creatinine	Elevated, reflects renal dysfunction Supportive, not predictive
Vahlensieck et al., 2015 [37]	Germany	57	5 years	CRP ≥ 15 mg/dL	Elevated in most cases, especially elderly Limited specificity for sepsis
Stokes et al., 2021 [26]	USA	147	NR	PSEP	Elevated early in infection and severe AOP Rises faster than PCT Correlates with AKI and sepsis

CRP = C-reactive protein; WBC = white blood cells; AOP = acute and obstructive pyelonephritis; NLR = Neutrophil-to-Lymphocyte Ratio; PLR = Platelet-to-Lymphocyte Ratio; MLR = Monocyte-to-Lymphocyte Ratio; qSOFA = quick Sepsis-Related Organ Failure Assessment; SOFA = Sequential Organ Failure Assessment; IL-6 = interleukin-6; NGAL = Neutrophil Gelatinase-Associated Lipocalin; PCT = procalcitonin; PSEP = presepsin; AKI = acute kidney injury; NR = not reported.

**Table 6:** Microbiological spectrum and antimicrobial resistance in APN/AOP.

Reference	Country	No. of Patients	Study Period	Pathogen/Group	Frequency/Resistance Pattern
Jang et al., 2022 [23]	Multiple (Asia, Europe, Americas)	various	2014–2017	*Escherichia coli*	60–95% community-acquired; ESBL 20–70%; high FQ resistance
Hyun et al., 2019 [29]	South Korea	NR	2018–2019	*Klebsiella pneumoniae*	10–20% overall; ESBL ~65% in some cohorts; sensitive to ciprofloxacin/gentamicin; Important in obstructive cases
Cornejo-Dávila et al., 2015 [36]	Mexico	150	2014–2015	*E. coli* ESBL and mixed *Enterobacteriaceae*	High MDR rates; 65% ESBL-positive *E. coli*
				MDR/ESBL distribution	Cross-resistance to cephalosporins, TMP-SMX, FQ; lower to aminoglycosides; carbapenem resistance rare
Gottlieb et al., 2025 [38]	Global/multicenter	NR	2016–2024	*Pseudomonas, Enterococcus*	Healthcare-associated High resistance in ICU settings Intrinsic β-lactam resistance
				*Candida* spp.	Catheterized/immunocompromised; Possible azole resistance
Rudrabhatla, 2018 [32]	Multiple	NR	2016–2017	*E. coli*	Predominant pathogen Cross-resistance common
EAU 2025 Guideline, 2025 [10]	Europe	NR	2025	*Proteus, Enterobacter, Citrobacter* spp.	Frequently MDR Linked to stones, recurrent infections

ESBL = extended-spectrum beta-lactamase; FQ = fluoroquinolone; MDR = multi-drug-resistant; TMP-SMX = Trimethoprim/sulfamethoxazole; *E. coli* = *Escherichia coli*; ICU = intensive care unit; NR = not reported.

**Table 7:** Imaging findings and radiological predictors in APN/AOP.

Reference	Country	No. of Patients	Study Period	Modality	Typical Findings	Performance/Clinical Significance
Enikeev et al., 2017 [34]	NR	207	NR	Ultrasound (B-mode)	Renal enlargement, parenchymal thickening, ‘prominent renal pyramid’	Abnormal in ~57.9% of cases; limited sensitivity; useful for bedside evaluation
	NR	NR	NR	Color Doppler US	Increased global perfusion; focal avascular areas in abscess/carbuncle	Improves detection of abscesses and destructive lesions
Enikeev et al., 2017 [34]	Russia (probable)	NR	2017	CT (non-contrast and contrast-enhanced)	Renal enlargement, striated nephrogram, hypodense areas, fat stranding, gas	Sensitivity 71.7–100% for complications; guides drainage decisions
Abi Tayeh et al., 2022 [39]	Lebanon	NR	2021–2022	US and CT in pyonephrosis	Hydronephrosis, fluid-pus levels, echogenic debris, thickened walls	Pelvic wall thickening sens. ~76% for pyonephrosis; gas = specific for infection
Hayano et al., 2024 [19]	Japan	NR	2024	Point-of-care Gram stain (adjunct)	Rapid urine Gram stain for empirical guidance	Aids empiric antibiotic choice; complements imaging
Pierce et al., 2019 [30]	USA	NR	NR	US (B-mode)	Abnormal in 57.9%—renal enlargement, medullary thickening, ‘Proeminent renal pyramid’ sign (25.6%)	Non-invasive Limited sensitivity (20.7% normal
				CT (contrast-enhanced)	Reduced enhancement, striated nephrogram Gas in collecting system, perirenal fat stranding	71.7–100% detection; guides drainage
Lee E.H. et al., 2019 [31]	South Korea	NR	NR	MRI	T2 hyperintense/T1 hypointense zones; Multiloculated abscess, cyst differentiation	Equal to CT in sensitivity/specificity
Yamamichi et al., 2018 [33]	Japan	NR	2015–2018	Predictors of obstruction	Stone > 5 mm, hydronephrosis, fat stranding, gas	Markers of severe AOP; Guide early stenting/intervention

APN = acute pyelonephritis; AOP = acute and obstructive pyelonephritis; US = ultrasound; CT = computed tomography; MRI = magnetic resonance imaging; NR = not reported.

**Table 8:** Therapeutic and interventional management in APN/AOP.

Aspect	Findings/Interventions	Key Outcomes/Implications
Empirical antibiotic therapy [23,38]	Cephalosporins, piperacillin–tazobactam, carbapenems, aminoglycosides	Broad-spectrum; de-escalate when possible
Resistance influence [38]	ESBL 20–70%; limits FQ, 3rd-gen cephalosporins	Stewardship essential
Empirical adequacy [29]	Higher for *K. pneumoniae* (96%) vs. *E. coli* (70.6%)	Reflects resistance variability
Optimal duration [32]	7-day non-FQ = 14-day; FQ ≤ 7 days equally effective	Short course safe, prevents resistance
Source control [36,37]	DJ stenting (76.6%), nephrostomy, abscess drainage (75%)	Essential for obstruction; improves survival
Surgical management [36,37]	Suprapubic catheter (49), deroofing (33), orchiectomy (14), cystolitholapaxy (11)	Tailored to complications
Supportive therapy [26,27]	Fluids, norepinephrine, insulin, corticosteroids	Adheres to sepsis protocols
Antimicrobial stewardship [23]	Prioritize β-lactam/β-lactamase inhibitors, carbapenems	Guided by resistance data

ESBL = extended-spectrum beta-lactamase; FQ = fluoroquinolone; *K. pneumoniae* = *Klebsiella pneumoniae*; DJ = double J.

**Table 9:** Comparative overview of the results.

Parameter	Findings in Current Study	Comparable Studies	Main Similarities/Differences
Clinical	Elderly and patients with DM at higher risk; atypical symptoms in elderly; need for early decompression	Chang et al. [35]; Lee et al. [28]; Yamamichi et al. [33]	Concordant findings; current study reinforces early intervention as survival determinant
Biological	PCT and PSEP superior to CRP; NLR, hypoalbuminemia, and thrombocytopenia predict septic shock	Baboudjian et al. [25]; Tambo et al. [27]; Truong et al. [18]	Strong concordance; adds local validation of biomarker thresholds
Microbiological	*E. coli* predominant (60–95%); ESBL 20–70%; MDR increasing	Jang et al. [23]; Hyun et al. [29]; Cornejo-Dávila et al. [36]	Matches global data; supports shift toward carbapenem and β-lactam/β-lactamase therapy
Imaging	US sensitivity ~58%; CT 71–100%; MRI useful for abscess differentiation	Enikeev et al. [34]; Abi Tayeh et al. [39]	Consistent diagnostic hierarchy; confirms CT as gold standard and MRI as adjunct

DM—Diabetes Mellitus; PCT = procalcitonin; PSEP = presepsin; CRP = C-reactive protein; NLR = Neutrophil-to-Lymphocyte Ratio; *E. coli* = *Escherichia coli*; ESBL = extended-spectrum beta-lactamase; MDR = multi-drug-resistant; US = ultrasound; CT computed tomography; MRI = magnetic resonance imaging.

**Table 10:** Main differences between AOP and APN.

Parameter	APN	AOP
Definition	Acute bacterial infection of the renal parenchyma and pelvis	Acute pyelonephritis occurring in the presence of urinary tract obstruction
Pathophysiology	Ascending infection from the lower urinary tract	Ascending infection combined with impaired urinary drainage
Common causes	Vesicoureteral reflux, urinary stasis, uropathogens	Urolithiasis, ureteral strictures, tumors, prostatic obstruction
Intraluminal pressure	Normal or mildly increased	Significantly increased, leading to renal parenchymal compression
Severity	Usually moderate to severe	More severe, rapidly progressive
Risk of sepsis	Present	Markedly increased
Response to Antibiotic-therapy	Often sufficient as monotherapy	Insufficient without relieving obstruction
Management	Prompt antimicrobial therapy	Emergency urinary decompression and antibiotherapy
Prognosis	Generally, favorable with treatment	Poorer if obstruction is not rapidly eliminated

APN—Acute Pyelonephritis; AOP—Acute Obstructive Pyelonephritis.

## Data Availability

The original contributions presented in this study are included in the article. Further inquiries can be directed to the corresponding author.

## Supplementary Materials

The following supporting information can be downloaded at: https://www.mdpi.com/article/10.3390/medicina62010222/s1, Table S1: PRISMA 2020 Checklist [52].

## Abbreviations

The following abbreviations are used in this manuscript:

AOP	Acute and Obstructive Pyelonephritis
APN	Acute Pyelonephritis
CFU	Colony-forming Unit
CRP	C-reactive Protein
CIs	Confidence Interval
CT	Computed Tomography
DJ	Double-J
DM	Diabetes Mellitus
ESBL	Extended-spectrum β-lactamase
MDR	Multi-Drug-Resistant
MRI	Magnetic Resonance Imaging
NLR	Neutrophil-to-lymphocyte Ratio
NOS	Newcastle–Ottawa Scale
PCN	Percutaneous Nephrostomy
PCT	Procalcitonin
PSEP	Presepsin
US	Ultrasound
UTIs	Urinary Tract Infections
